# Health insurance status and hearing aid utilization in U.S. older adults: A population-based cross-sectional study

**DOI:** 10.1371/journal.pone.0341570

**Published:** 2026-01-27

**Authors:** Kaitlin Hori, Albert Li, Diego E. Razura, John Parsons, Janet S. Choi

**Affiliations:** 1 Keck School of Medicine, University of Southern California, Los Angeles, California, United States of America; 2 Department of Otolaryngology-Head and Neck Surgery, Mayo Clinic Arizona, Phoenix, Arizona, United States of America; 3 Caruso Department of Otolaryngology-Head and Neck Surgery, University of Southern California, Los Angeles, California, United States of America; Universiti Malaya Fakulti Perubatan: University of Malaya Faculty of Medicine, MALAYSIA

## Abstract

**Background:**

The role of health insurance and its diverse hearing health benefits on hearing aid utilization is currently unknown. The objective of this study is to examine rates of ever and regular hearing aid (HA) use by insurance status in older U.S. adults.

**Methods:**

This cross-sectional study utilized data from the National Health and Nutrition Examination Survey (NHANES) (2005–2018). Older adults (≥65 years) with complete data on health insurance, audiometry, and hearing aid use (n = 3,172) were included. Eight combinatorial insurance categories were created and compared pairwise to the reference of Medicare only coverage. Outcomes included ever and regular hearing aid use.

**Results:**

Among older U.S. adults, 30.3% [95% CI:27.6%−33.2%] of those with audiometry-measured hearing loss reported ever using HAs while 22.9% [95% CI:20.3%−25.7%] reported regular HA use. Among older adults with hearing loss, those with military-related insurance (Tricare, VA and Champ-VA) had amongst the highest rates of ever and regular HA use (43.3% [95% CI:31.3%−56.2%] and 30.8% [95% CI:21.1%−42.5%], respectively). Ever HA use rates for individuals with Medicare and Medicaid was 30.8% [95% CI:27.8%−33.8%] and 17.7% [95% CI:11.9%−25.5%], respectively. In a multivariable model adjusting for demographics and hearing loss severity, individuals with military-related and military-related+Medicaid insurance were significantly more likely to report ever using HAs compared to those with Medicare only (OR 1.80, 95% CI:1.03–3.16; OR 20.38, 95% CI:1.07–386.84, respectively). Those with military-related insurance were more likely to report regular HA use (OR 2.17, 95% CI:1.16–4.09).

**Conclusion:**

In this nationally representative study of older U.S. adults, we found differences in ever and regular HA use rates by insurance status, even when adjusting for hearing loss, demographics, and comorbidities. Future research is warranted to investigate group-specific differences, including access to hearing care, hearing health benefits, and stigma, to better understand the facilitators and barriers to hearing aid use by insurance status.

## Introduction

Hearing loss is a significant public health concern and the leading sensory disorder among United States (U.S.) adults over 65 years old [1–4]. Untreated hearing loss is associated with adverse health outcomes, including reduced quality of life, and increased risk of cognitive decline and mortality [5–9]. It also imposes a substantial healthcare burden, with excess medical costs exceeding $3 billion [10–14].

Despite scientific consensus on the benefits of hearing aids (HA) for communication and quality of life, 60–90.3% of Americans with hearing loss do not use them [2,15]. Numerous barriers contribute to low utilization rates, with one major barrier being the high cost associated with obtaining them, including expenses for audiological diagnostics and follow-up visits [16–18]. The average cost of prescription HAs ranges from $2,000-$5,000, with out of pocket expenses depending on an individual’s insurance coverage [14,16,19,20].

Medicare Parts A and B currently do not cover HAs or fitting exams, while only certain Medicare Advantage (Part C) plans offer partial hearing care benefits [20–22]. Medicaid coverage for HAs varies by state, with coverage ranging from initial assessments and fittings to ongoing expenses such as batteries, repairs, replacements and follow-up appointments [23,24]. In comparison, eligible Veterans Affairs (VA) and Champ-VA insurance beneficiaries can access benefits that either fully cover hearing health related costs or provide low-cost options [20,25,26]. Similarly, TRICARE insurance provides HA coverage and related services for eligible active-duty service members, career military retirees and their families [20,27]. Meanwhile, private insurance plans vary in their HA coverage depending on plan type and state regulations [28,29].

In this study, we aim to understand discrepancies in HA utilization among older U.S. adults (aged 65 and above) across various insurance coverage, including Medicare, Medicaid, private, military-related (Tricare, VA, and Champ-VA), and no insurance. Using a nationally representative sample of older U.S. adults, we examined the associations between different combinatorial insurance groups (Eight categories, compared pairwise to Medicare-only as the reference) and both ever and regular HA use in multivariable models adjusting for audiometry-measured hearing loss severity and demographic and clinical factors.

## Methods

This is a cross-sectional study based on five cohorts of older adults who participated in the National Health and Nutrition Examination Survey (NHANES) (2005–2006, 2009–2010, 2011–2012, 2015–2016, 2017–2018). Data was accessed on August 13, 2024. Data was publicly available and deidentified, so the need for individual patient consent was waived. The study protocol was reviewed by the University of Southern California Institutional Review Boards and deemed exempt (UP-20–01447).

NHANES is an ongoing data collection by the U.S. Centers for Disease Control and Prevention to assess the nutrition and health status of the non-institutionalized, non-active duty military U.S. population. Each study cycle uses a stratified, multistage probability sampling design with selective oversampling of low income and racial minority individuals. Sampling weights are used to analyze the survey to create results applicable to and representative of the diverse U.S. population. Our cohort consisted of participants in the five NHANES cohorts aged 65 years or older with complete data on audiometry-measured hearing, HA use, and insurance status. Hearing loss and HA use were assessed as survey-weighted proportions of the population and in relation to insurance status.

### Audiometry

Audiometry testing was conducted by examiners trained by an audiologist from the National Institute for Occupational Safety & Health (NIOSH) [30]. An examiner determined the air conduction hearing threshold for each ear without a HA in a sound-isolated room. Testing was conducted using the automated testing mode of the audiometer Interacoustics Model AD226 (Assens, Denmark). Audiometric equipment and sound booths (same brands and models) were used, and data was captured electronically and uploaded automatically. Daily equipment calibration and monitoring of ambient noise levels using a sound level meter were performed. Speech-frequency pure-tone average (PTA) was calculated for each ear based on thresholds at 0.5 kHz, 1 kHz, 2 kHz, and 4 kHz. Audiometry-measured hearing loss was defined as speech-frequency PTA at 25 dB HL (hearing level) or greater in the better hearing ear, as defined by the World Health Organization (WHO) [31]. Hearing loss was further categorized into mild, moderate, and severe to profound hearing loss based on the WHO grading (mild, 25 to <40 dB HL; moderate, 40 to <60 dB HL; severe to profound, ≥ 60 dB HL).

### Hearing aid use

HA use was determined at the time of NHANES participation and categorized into two outcomes based on audiometry questions in each cohort: (1) ever use (never vs. ever use) and (2) regular use (never vs. non-regular vs. regular use). Participants were categorized as ever HA users if they responded “YES” to “Ever worn a HA?” or “Now use a HA?”. Never HA users responded “NO” to the questions above. Participants who reported hearing amplifier or cochlear implant use were excluded.

Regular HA use was based on questions on frequency of use, which varied by cycle [9]. Participants were considered regular HA users when reporting: (1) wearing HAs for at least five hours a week for the past year (2005−06, 2009−10); (2) HA use at least half of the time, usually or always for the past year (vs seldom or never; 2011−12, 2015−16); (3) One hour or more of HA use per day for the last two weeks (vs less than one hour; 2017−18). Participants who reported ever HA use but did not meet criteria for regular use were considered non-regular users. NHANES questions to define HA use are summarized in S1 Table.

### Insurance status

Insurance groups analyzed were Medicare, Medicaid, private, military-related (including Tricare, VA and Champ-VA), and no insurance. As part of NHANES, participants selected all applicable insurance coverage at the time of survey completion. Insurance types were recoded into binary variables indicating whether participants had the insurance or not. Combinatorial insurance groups were created to account for participants with multiple insurance coverages. For descriptive statistics, participants were categorized into non-mutually exclusive groups for all insurances selected. Weighted proportions were used to estimate the distribution of each insurance group among the U.S. population. For logistic regression analyses, eight combinatorial groups were created and compared pairwise to Medicare-only coverage (reference group). The groups were: military-related, Medicaid, private, No Insurance, military+Medicaid, military+private, Medicaid+private and military+private+Medicaid. Since this study focuses on older U.S. adults, where Medicare coverage is common, individuals in these groups could also have Medicare coverage.

### Other covariates

Demographics and medical history were self-reported and obtained from interview data. Sex was categorized into male or female. Race and ethnicity were classified as “White”, “Black”, “Hispanic”, or “Others”. Marital status was categorized into “Married or Living with Partner”, “Widowed”, “Divorced or Separated”, and “Never Married”. Education was categorized into “Less than High School”, “High School Graduate”, and “Some College or more”. Income was stratified into “<20K”, “20-44.9K”, “45-74.9K”, “>75K” and “Refused/Don’t Know”. Medical comorbidities included hypertension, diabetes, and stroke, which were coded into binary variables indicating having the illness or not. Smoking status was coded into a categorical variable indicating “Never”, “Former” or “Current” use.

### Statistical analysis

Categorical data was summarized using counts and percentages. Continuous data was reported with means and standard deviations. Baseline characteristics were compared using Pearson’s chi-squared test for categorical variables and the Student’s t-test for continuous variables. Survey weights were applied to estimate proportions of insurance groups, hearing loss and HA use that are generalizable to the U.S. population.

The association between hearing loss and insurance status was examined with hearing loss coded as a binary variable using multivariable logistic regression models and as a continuous variable using multivariable regression analysis sequentially adjusted for age, demographics and medical history. The association between HA use and insurance status was evaluated with multivariable logistic regression models sequentially adjusting for the same variables mentioned above in addition to hearing loss severity (continuous speech-frequency PTA in the better hearing ear). Two HA use outcomes were analyzed against insurance status: (1) ever use comparing ever use to never use; (2) regular use comparing regular use to never use. To address potential overlap in insurance categories, sensitivity analysis was conducted using 15 mutually exclusive insurance groupings. Logistic regression models were run with the same variables as the main analysis. All analyses were conducted with STATA (version 18.0, StataCorp. LLC), and p-values <0.05 were considered statistically significant.

## Results

The characteristics of the study cohort (n = 3,172) organized by hearing loss and HA use are summarized in Table 1. Table 2 presents a survey-weighted analysis of hearing loss prevalence across different insurance groups, categorizing hearing levels as normal, mild, moderate, and severe or profound. Multivariable logistic and regression analysis comparing hearing loss as both a binary and continuous variable against insurance groups showed no significant association between hearing loss and insurance status.

**Table 1:** Summary of study participant characteristics, NHANES (n = 3172).

	Total	Hearing Status		Hearing Aid Use			
				Non-Hearing Aid Users	Ever Hearing Aid Users		
		Hearing Loss	No Hearing Loss		All Hearing Aid Users	Non-Regular Hearing Aid Users	Regular Hearing Aid Users
Unweighted Number (n)	3172	1862	1310	1359	503	156	347
**Age, mean (SD)**	74.5 (0.10)	76.3 (0.12)	71.9 (0.14)	75.9 (0.14)	77.5 (0.21)	77.6 (0.38)	77.5 (0.25)
**Gender, no. (%)**							
Male	1600 (50.4)	1042 (56.0)	558 (42.6)	718 (52.8)	324 (64.4)	107 (68.6)	217 (62.5)
Female	1572 (49.6)	820 (44.0)	752 (57.4)	641 (47.2)	179 (35.6)	49 (31.4)	130 (37.5)
**Race, no. (%)**							
White	1886 (59.5)	1264 (67.9)	622 (47.5)	870 (64.0)	394 (78.3)	104 (66.7)	290 (83.6)
Black	552 (17.4)	221 (11.9)	331 (25.3)	183 (13.5)	38 (7.6)	16 (10.3)	22 (6.3)
Hispanic	504 (15.9)	261 (14.0)	243 (18.6)	211 (15.5)	50 (9.9)	27 (17.3)	23 (6.6)
Others	230 (7.3)	116 (6.2)	114 (8.7)	95 (7.0)	21 (4.2)	9 (5.8)	12 (3.5)
**Marital Status, no. (%)**							
Married	1760 (55.5)	1007 (54.1)	753 (57.5)	713 (52.5)	294 (58.5)	91 (58.3)	203 (58.5)
Widowed	876 (27.6)	577 (31.0)	299 (22.8)	420 (30.9)	157 (31.2)	42 (26.9)	115 (33.1)
Divorced/ Separated	417 (13.2)	223 (12.0)	194 (14.8)	185 (13.6)	38 (7.6)	18 (11.5)	20 (5.8)
Never Married	119 (3.8)	55 (3.0)	64 (4.9)	41 (3.0)	14 (2.8)	5 (3.2)	9 (2.6)
**Education, no. (%)**							
Less than High School Grad	969 (30.6)	632 (34.0)	337 (25.7)	494 (36.4)	138 (27.4)	61 (39.1)	77 (22.1)
High School Grad	768 (24.2)	470 (25.2)	298 (22.8)	355 (26.1)	115 (22.9)	25 (16.0)	90 (26.0)
Some College or More	1429 (45.1)	756 (40.6)	673 (51.4)	507 (37.3)	249 (49.5)	70 (44.9)	179 (51.6)
**Income, no. (%)**							
<20k	840 (26.5)	533 (28.6)	307 (23.4)	432 (31.8)	101 (20.1)	48 (30.8)	53 (15.3)
20-44.9K	1044 (32.9)	649 (34.9)	395 (30.2)	468 (34.4)	181 (36.0)	51 (32.7)	130 (37.5)
45-74.9K	536 (16.9)	293 (15.7)	243 (18.6)	201 (14.8)	92 (18.3)	26 (16.7)	66 (19.0)
>75K	470 (14.8)	235 (12.6)	235 (17.9)	147 (10.8)	88 (17.5)	20 (12.8)	68 (19.6)
Refused or Don’t Know	282 (8.9)	152 (8.2)	130 (9.9)	111 (8.2)	41 (8.2)	11 (7.0)	30 (8.7)
**Comorbidities, no. (%)**							
Diabetes	823 (26.0)	490 (26.3)	333 (25.4)	367 (27.0)	123 (24.5)	44 (28.2)	79 (22.8)
Hypertension	1978 (62.4)	1146 (61.6)	832 (63.5)	852 (62.7)	294 (58.5)	97 (62.2)	197 (56.8)
Stroke	304 (9.6)	207 (11.1)	97 (7.4)	147 (10.8)	60 (11.9)	18 (11.5)	42 (12.1)
Current Cigarette Smoker	309 (9.74)	156 (8.4)	153 (11.7)	130 (9.6)	26 (5.2)	12 (7.7)	14 (4.0)
Former Cigarette Smoker	1332 (42.0)	829 (44.5)	503 (38.4)	581 (42.8)	248 (49.3)	76 (48.7)	172 (49.6)

No. = number of participants.

**Table 2:** Survey-Weighted Prevalence of Hearing Loss by Insurance Group.

	Normal Hearing		Mild Hearing Loss		Moderate Hearing Loss		Severe or Profound Hearing Loss	
Insurance	Proportion	95% CI	Proportion	95% CI	Proportion	95% CI	Proportion	95% CI
Military	0.39	(0.31, 0.47)	0.33	(0.25, 0.41)	0.25	(0.18, 0.33)	0.04	(0.02, 0.07)
Medicare	0.45	(0.41, 0.49)	0.31	(0.29, 0.34)	0.20	(0.18, 0.23)	0.04	(0.03, 0.04)
Medicaid	0.47	(0.40, 0.54)	0.28	(0.22, 0.35)	0.21	(0.16, 0.27)	0.04	(0.03, 0.08)
Private	0.46	(0.42, 0.51)	0.31	(0.28, 0.34)	0.20	(0.17, 0.23)	0.03	(0.03, 0.04)
None	0.40	(0.25, 0.55)	0.39	(0.21, 0.59)	0.12	(0.06, 0.24)	0.10	(0.03, 0.32)

Note: Insurance groups were not mutually exclusive. For descriptive statistics, participants could be part of multiple insurance groups.

Hearing loss was categorized based on audiometry exam into mild, moderate, and severe to profound hearing loss as follows (mild, 25 to <40 dB HL; moderate, 40 to <60 dB HL; severe to profound, ≥ 60 dB HL).

**Fig 1:**
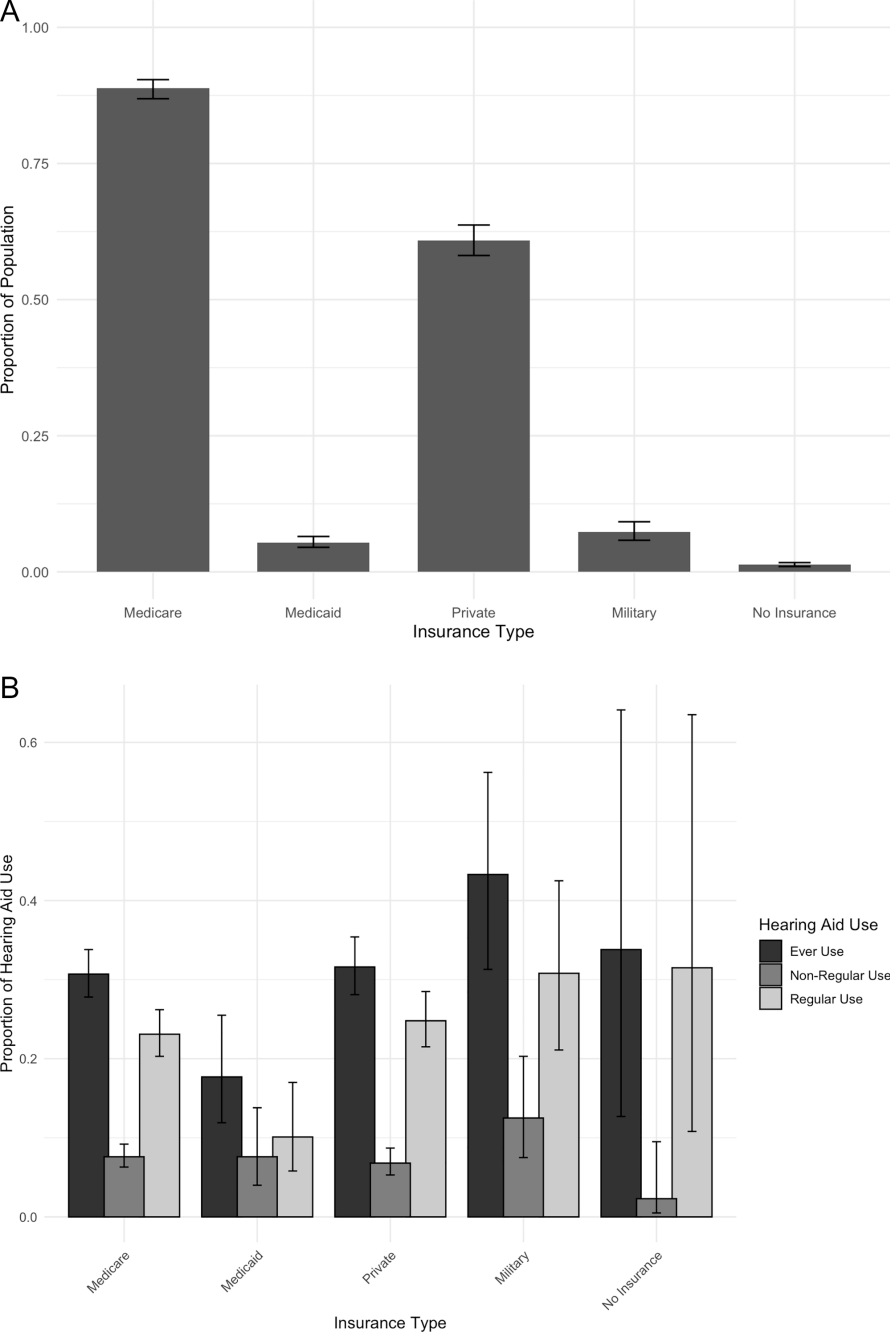
A. Survey-Weighted Proportion of Population with Each Insurance Type. Note: Insurance groups are not mutually exclusive. B. Survey-weighted proportions of hearing aid use by insurance status. Note: Insurance groups are not mutually exclusive.

Fig 1A shows the results of a survey weighted analysis conducted to determine the proportions of non-mutually exclusive insurance coverage. 88.8% [95% CI:86.9%−90.4%] of the population had Medicare coverage, 5.4% [95% CI:4.5%−6.5%] had Medicaid, 60.9% [95% CI:58.1%−63.7%] had private, 7.3% [95% CI:5.8%−9.2%] had military-related insurance, and 1.3% [95% CI:0.98%−1.7%] had no insurance coverage.

Among older U.S. adults, 30.3% [95% CI:27.6%−33.2%] of those with audiometry-measured hearing loss reported ever HA use and 22.9% [95% CI:20.3%−25.7%] reported regular use. Fig 1B shows survey-weighted proportions of HA utilization by those with hearing loss amongst the non-mutually exclusive insurance groups (Proportions in S2 Table). Those with military-related insurance had amongst the highest ever and regular HA use rates (43.3% [95% CI:31.3%−56.2%] and 30.8% [95% CI:21.1%−42.5%], respectively).

The association between ever and regular HA use and insurance status was examined using eight combinatorial insurance groups compared pairwise to Medicare only, as detailed in Methods (Tables 3 and 4). When adjusted for demographics, hearing loss, and medical comorbidities, those with military-related coverage and military+Medicaid insurance were more likely to ever use HAs compared to those with Medicare alone (OR 1.80, 95% CI:1.03–3.16; OR 20.38, 95% CI:1.07–386.84, respectively). Those with military-related insurance were more likely to report regular HA use compared to those with Medicare alone (OR 2.17, 95% CI:1.16–4.09). There were no significant differences in ever or regular HA use between individuals with Medicare only and those with private insurance (Tables 3 and 4). Sensitivity analysis, with mutually exclusive insurance groups compared to Medicare alone, produced results largely consistent with the main analysis (S3 and S4 Tables). Those with military+Medicare reported greater regular HA use compared to those with Medicare alone (OR 2.10, 95% CI:1.07–4.11). Groups with military-related insurance generally demonstrated greater reports of both ever and regular HA use.

**Table 3:** Logistic regression analysis examining the association between insurance coverage and ever hearing aid use.

Insurance Coverage	Cohort Size (n)	Unadjusted		Multivariable Models					
		OR (95% CI)	P-Value	Model 1† OR (95% CI)	P-Value	Model 2‡ OR (95% CI)	P-Value	Model 3†† OR (95% CI)	P-Value
Medicare	954	1 (ref)	–	1 (ref)	–	1 (ref)	–	1 (ref)	–
Military (can include those with Medicare)	179	1.95* (1.26-3.02)	0.003	2.06* (1.22-3.50)	0.007	1.83* (1.05-3.20)	0.034	1.80* (1.03-3.16)	0.040
Medicaid (can include those with Medicare)	250	0.46* (0.27-0.80)	0.006	0.41* (0.22-0.79)	0.007	0.57 (0.29-1.13)	0.106	0.57 (0.29-1.14)	0.111
Private (can include those with Medicare)	1,571	1.27 (1.00-1.61)	0.052	1.57* (1.16-2.11)	0.003	1.33 (0.98-1.82)	0.068	1.32 (0.96-1.80)	0.083
No Insurance	120	0.43* (0.20-0.92)	0.030	0.41 (0.15-1.07)	0.069	0.52 (0.18-1.46)	0.211	0.53 (0.18-1.49)	0.227
Military & Medicaid (can include those with Medicare)	6	3.05 (0.19-49.09)	0.431	30.35* (1.74-528.05)	0.019	23.46* (1.25-439.49)	0.035	20.38* (1.07-386.84)	0.045
Military & Private (can include those with Medicare)	56	1.83 (0.99-3.39)	0.054	2.29* (1.11-4.73)	0.025	1.71 (0.78-3.74)	0.180	1.81 (0.82-4.00)	0.144
Medicaid & Private (can include those with Medicare)	33	0.95 (0.34-2.65)	0.927	0.86 (0.25-2.94)	0.808	0.93 (0.27-3.22)	0.909	0.89 (0.25-3.12)	0.850
Military, Private & Medicaid (can include those with Medicare)	79	1.65 (0.93-2.94)	0.089	1.91 (0.97-3.78)	0.062	1.53 (0.75-3.15)	0.245	1.61 (0.78-3.34)	0.199

† Model 1: Adjusted for Age and Hearing Loss.

‡ Model 2: Adjusted for Age, Hearing Loss, Gender, Race, Education, and Income.

†† Model 3: Adjusted for Age, Hearing Loss, Gender, Race, Education, Income, Hypertension, Diabetes, Stroke & Smoking.

* p < 0.05.

**Table 4:** Logistic regression analysis examining the association between insurance coverage and regular hearing aid use.

Insurance Coverage	Cohort Size (n)	Unadjusted		Multivariable Models					
		OR (95% CI)	P-Value	Model 1† OR (95% CI)	P-Value	Model 2‡ OR (95% CI)	P-Value	Model 3†† OR (95% CI)	P-Value
Medicare only	954	1 (ref)	–	1 (ref)	–	1 (ref)	–	1 (ref)	–
Military (can include those with Medicare)	179	2.26* (1.38-3.69)	0.001	2.28* (1.27-4.11)	0.006	2.18* (1.17-4.08)	0.015	2.17* (1.16-4.09)	0.016
Medicaid (can include those with Medicare)	250	0.34* (0.16-0.73)	0.006	0.27* (0.11-0.63)	0.003	0.43 (0.17-1.08)	0.073	0.48 (0.19-1.21)	0.119
Private (can include those with Medicare)	1,571	1.46* (1.11-1.94)	0.007	1.75* (1.23-2.49)	0.002	1.41 (0.97-2.04)	0.071	1.39 (0.96-2.02)	0.084
No Insurance	120	0.42 (0.16-1.08)	0.073	0.42 (0.13-1.39)	0.154	0.64 (0.18-2.23)	0.481	0.63 (0.18-2.19)	0.470
Military & Medicaid (can include those with Medicare)	6	–	–	–	–	–	–	–	–
Military & Private (can include those with Medicare)	56	1.93 (0.95-3.91)	0.069	1.90 (0.82-4.39)	0.133	1.41 (0.57-3.49)	0.455	1.52 (0.61-3.82)	0.368
Medicaid & Private (can include those with Medicare)	33	0.90 (0.26-3.17)	0.874	0.76 (0.17-3.31)	0.715	1.02 (0.24-4.33)	0.984	0.99 (0.22-4.35)	0.989
Military, Private & Medicaid (can include those with Medicare)	79	1.69 (0.86-3.32)	0.125	1.62 (0.73-3.58)	0.235	1.31 (0.57-3.04)	0.525	1.42 (0.60-3.33)	0.422

† Model 1: Adjusted for Age and Hearing Loss.

‡ Model 2: Adjusted for Age, Hearing Loss, Gender, Race, Education, and Income.

†† Model 3: Adjusted for Age, Hearing Loss, Gender, Race, Education, Income, Hypertension, Diabetes, Stroke & Smoking.

* p < 0.05.

Military & Medicaid unable to analyze due to collinearity.

## Discussion

In this nationally representative study, approximately half of U.S. adults over 65 years old demonstrated audiometry-measured hearing loss. Among those with hearing loss, 30.3% reported ever HA use and 22.9% reported regular use. There were discrepancies in HA utilization based on insurance status. Individuals with military+Medicaid generally exhibited higher rates of ever HA use, while those with military-related insurance had higher rates of ever and regular HA use. There were no significant differences in HA utilization rates between individuals with private insurance and Medicare only.

The prevalence of ever HA use (30.3%) in this study aligns with previous reports of 22–30% among older U.S. adults [22,32]. These generally low rates of HA utilization have been consistently documented across studies [33–36]. Numerous factors contribute to this underutilization, with cost being one of the major barriers, particularly for individuals with low socioeconomic status [37,38]. One study found that a bundled cost of $2,500 for HAs were unaffordable for three-fourths of those with hearing loss, while another identified insurance coverage as a key facilitator for HA use [17,39].

In this study, older adults with military-related insurance reported significantly higher rates of ever and regular HA use compared to those with Medicare alone even after adjusting for severity of hearing loss, demographics including socioeconomic status and medical comorbidities. These results suggest that enhanced coverage, such as that provided by military-related insurance, may encourage both the initial trial and sustained use of HAs. Military-related insurance (VA, Tricare and Champ-VA) provides relatively more comprehensive coverage for evaluation, fitting and repairs of HAs [25,27]. In contrast, Medicare Parts A and B do not provide any HA coverage and Medicaid’s hearing related coverage varies by state with only 28 states providing some level of coverage [20,21,24]. Based on these coverages, our findings suggest that expanding insurance coverage to remove financial barriers may help to improve both uptake and long-term adherence to HAs. Due to the survey’s inclusion of a single binary question assessing military-related insurance coverage (including VA, TRICARE, and Champ-VA), separate analyses by each plan could not be performed. Notably, military-related insurance covers not only veterans but also non-veteran family members. However, in this cohort, most individuals with military-related insurance (79.5%) reported having served on active duty.

While expanded insurance coverage, as is offered by military-related insurance, may alleviate the financial barrier to obtaining HAs, there are still many other factors that influence HA use. One is the perceived stigma associated with hearing loss and HA use. Previous research amongst the elderly and veteran populations found that this stigma can negatively impact self-perception and relationships, which can delay acceptance of hearing loss, contribute to low utilization of audiology services and influence when and where HAs are used [40,41]. Furthermore, perceived stigma may influence self-reports of hearing loss and HA use. In this study, hearing loss was determined by audiometric data, minimizing the potential overestimation of HA use associated with under-report of hearing loss among older adults. However, HA use was self-reported and may have been influenced by stigma, as participants’ perceptions of HAs could affect their responses. Veterans and active duty personnel are more likely than civilians to experience combat-related auditory trauma, leading to hearing loss that requires HAs [42,43]. Consequently, HA use may be more accepted among the military-insured population, beyond the influence of lower costs driving higher HA adoption rates. Future studies that examine participants’ specific attitudes toward HAs will provide deeper insight into key barriers and facilitators across different insurance groups.

Prior studies on the association between HA use and insurance found varying results. A study based on the Health and Retirement Study, which included participants aged 55 and older with self-reported hearing loss, found greater odds of HA use among those with VA health insurance, consistent with our findings [36]. Another study of adults aged 65 and older utilizing the National Health Interview Survey, which included self-reported hearing loss, similarly found that those with military insurance alone or combined with Medicare had higher rates of ever HA use compared to individuals with Medicare alone [44]. In contrast, two prior NHANES studies found no significant association between insurance status and HA use [18,34]. These discrepancies are likely attributable to differences in age criteria and the lack of further categorization of insurance status due to limited sample size in the prior NHANES studies. For instance, these two studies did not independently analyze military-related insurance. Our study builds on prior research by adjusting analyses for audiometry-measured hearing loss and offering a comprehensive perspective on both ever and regular HA use. This represents the first nationally representative study to examine both uptake and continued use of HAs.

While residual confounders, such as awareness of hearing loss and stigma associated with HAs persist, findings from this study suggest that insurance status, along with its hearing health benefits may play a role in influencing HA acquisition and use. As the aging population grows, rates of hearing loss are expected to simultaneously expand [32,45]. There is growing literature demonstrating the detrimental effects that hearing loss can have on social, mental, and cognitive health [5–8]. These outcomes cause financial strain on the U.S. healthcare system and should motivate various stakeholders to discuss policy changes related to access and cost of hearing healthcare [10–14]. Future research can contribute to these discussions by examining how insurance coverage modifications influence HA trials and utilization. It can also identify factors that affect adherence and long-term use, especially among individuals with sufficient financial coverage. Additionally, payment models for hearing healthcare, such as comprehensive coverage, bundled services, and fee-for-service, along with the integration of technologies such as telehealth, remote audiology services, and over-the-counter HAs, should be explored for their cost-effectiveness in promoting hearing health [46,47].

Our study has limitations. As a cross-sectional analysis, there is limited insight into temporal and causal relationships. Insurance status may have changed over time, and the reported insurance at the time of the survey may not have impacted HA utilization. Information on insurance status and HA use was self-reported, which introduces the potential for reporting and recall bias. Furthermore, NHANES does not capture a comprehensive listing of insurance types and plan details. Instead, coverage categories are often aggregated, as with military-related insurance, limiting the ability to meaningfully differentiate between levels of coverage. Relevant data on hearing loss and HA use were unavailable, such as the duration of hearing loss or HA use, severity of hearing loss at the time of HA acquisition, type of HA used, and satisfaction with HAs. Participants who reported use of hearing amplifiers or cochlear implants were excluded from the cohort. However, some may have previously used hearing aids, which could potentially confound the analysis. NHANES modified survey questions over time for certain variables, such as HA use, which may have influenced how cohorts reported their HA usage. For insurance status, categorizing multiple groups created small sample sizes, limiting the ability to conduct meaningful analysis. Speech audiometry, such as word recognition scores, which provide a more nuanced understanding of HA candidacy, was not available in this study. Despite these limitations, this study represents the largest population-based analysis of older adults that incorporates audiometry-measured hearing data and a comprehensive categorization of insurance status.

## Conclusion

This study based on a nationally representative sample of older U.S. adults found discrepancies in HA use based on insurance status. Those with military-related insurance had higher rates of ever and regular HA use, while those with military+Medicaid coverage exhibited higher rates of ever use compared to those with Medicare alone. Future research is needed to investigate how variables such as access to hearing health benefits influence HA use among older adults.

## Supporting information

S1 Table NHANES Questions to Define Hearing Aid Users. (PDF)

S2 Table Survey Weighted Proportions of Hearing Aid Use by Non-Mutually Exclusive Insurance Groups. (PDF)

S3 Table Logistic regression analysis examining the association between mutually exclusive insurance coverage and ever hearing aid use. (PDF)

S4 Table Logistic regression analysis examining the association between mutually exclusive insurance coverage and regular hearing aid use. (PDF)
