# Supplementary material for: Health insurance status and hearing aid utilization in U.S. older adults: A population-based cross-sectional study
Source: PLoS One. 2026 Jan 27;21(1):e0341570. doi: 10.1371/journal.pone.0341570 (PMC12843536; doi:10.1371/journal.pone.0341570)
Supplement: S1 Table — (PDF) [file pone.0341570.s001.pdf]

S1 Table. NHANES Questions to Define Hearing Aid Users

NHANES Cohort	NHANES Question #	NHANES Question	Never Use	Ever Use	Regular Use	Non-Regular Use
2005-06 2009-10	AUQ150	"Ever worn a hearing aid"	No	Yes	Yes	Yes
	AUQ171	"Worn Hearing aid 5 hours a week?" (For the past year)	N/A	N/A	Yes	No
2011-12 2015-16	AUQ146	"Ever worn hearing aid/cochlear implant"	No	Yes	Yes	Yes
	AUD148	"Hearing Aid or Cochlear Implant?"	N/A	Hearing Aid	Hearing Aid	Hearing Aid
	AUQ152	"Past year how often worn hearing aid?"	N/A	N/A	Always, Usually or About half the time	Seldom or Never
2017-18	AUQ147	"Now use hearing aid/amplifier/implant"	No	Yes	Yes	Yes
	AUQ149a	"Now use a hearing aid"	N/A	Yes	Yes	Yes
	AUQ 149b	"Now use a personal sound amplifier"	N/A	No	No	No
	AUQ149c	"Now have a cochlear implant	N/A	No	No	No
	AUQ153	"Past 2 weeks how often worn hearing aid"	N/A	N/A	1-3 hours a day, 4-7 hours a day, 8 or more hours a day	Less than 1 hour a day
	AUQ630	"Ever worn hearing aid/amplifier/cochlear implant" (Only asked to those who answered "No" to AUQ147)	No	Yes	No	Yes
