# Supplementary material for: Health insurance status and hearing aid utilization in U.S. older adults: A population-based cross-sectional study
Source: PLoS One. 2026 Jan 27;21(1):e0341570. doi: 10.1371/journal.pone.0341570 (PMC12843536; doi:10.1371/journal.pone.0341570)
Supplement: S2 Table — (PDF) [file pone.0341570.s002.pdf]

S2 Table. Survey Weighted Proportions of Hearing Aid Use by Non-Mutually Exclusive Insurance Groups

Hearing Aid Use Categorization	Insurance Groups	Proportion of Insurance Group with Hearing Aid Use (%)	95% Confidence Interval
Ever Hearing Aid Use	Medicare	30.7	27.8%-33.8%
	Medicaid	17.7	11.9%-25.5%
	Private	31.6	28.1%-35.4%
	Military	43.3	31.3%-56.2%
	None	33.8	12.7%-64.1%
Regular Hearing Aid Use	Medicare	23.1	20.3%-26.2%
	Medicaid	10.1	5.8%-17.0%
	Private	24.8	21.5%-28.5%
	Military	30.8	21.1%-42.5%
	None	31.5	10.8%-63.5%
Non-Regular Hearing Aid Use	Medicare	7.6	6.3%-9.2%
	Medicaid	7.6	4.0%-13.8%
	Private	6.8	5.3%-8.7%
	Military	12.5	7.5%-20.3%
	None	2.3	0.5%-9.5%
