# Supplementary material for: Health insurance status and hearing aid utilization in U.S. older adults: A population-based cross-sectional study
Source: PLoS One. 2026 Jan 27;21(1):e0341570. doi: 10.1371/journal.pone.0341570 (PMC12843536; doi:10.1371/journal.pone.0341570)
Supplement: S3 Table — (PDF) [file pone.0341570.s003.pdf]

S3 Table. Logistic regression analysis examining the association between mutually exclusive insurance coverage and ever hearing aid use

Insurance Coverage	Cohort Size (n)	Unadjusted		Multivariable Models					
		OR (95% CI)	P-Value	Model 1† OR (95% CI)	P-Value	Model 2‡ OR (95% CI)	P-Value	Model 3†† OR (95% CI)	P-Value
Medicare only	954	1 (ref)	-	1 (ref)	-	1 (ref)	-	1 (ref)	-
Military only	24	2.29 (0.78-6.71)	0.132	2.84 (0.82-9.87)	0.101	2.59 (0.70-9.58)	0.154	2.65 (0.71-9.84)	0.147
Medicaid only	45	0.46 (0.13-1.56)	0.212	0.32 (0.08-1.37)	0.126	0.37 (0.08-1.78)	0.215	0.39 (0.08-1.81)	0.228
Private only	216	0.87 (0.52-1.45)	0.598	1.26 (0.68-2.31)	0.463	1.14 (0.61-2.11)	0.688	1.12 (0.61-2.09)	0.711
Military & Medicare	155	1.91* (1.20-3.03)	0.006	1.97* (1.12-3.48)	0.019	1.72 (0.97-3.07)	0.064	1.73 (0.97-3.07)	0.064
Medicaid & Medicare	205	0.46* (0.26-0.84)	0.011	0.40* (0.20-0.80)	0.010	0.62 (0.31-1.30)	0.207	0.62 (0.30-1.30)	0.204
Private & Medicare	1,355	1.32* (1.04-1.68)	0.025	1.56* (1.16-2.10)	0.003	1.34 (0.99-1.82)	0.060	1.33 (0.98-1.81)	0.068
Military & Medicaid	1	-	-	-	-	-	-	-	-
Military & Private	8	2.29 (0.51-10.35)	0.282	3.05 (0.44-21.44)	0.261	2.73 (0.37-20.0)	0.323	2.65 (0.36-19.35)	0.338
Medicaid & Private	10	1.02 (0.20-5.10)	0.984	1.04 (0.16-6.94)	0.966	1.14 (0.17-7.76)	0.895	1.12 (0.17-7.59)	0.904
Medicare, Private & Military	48	1.76 (0.91-3.42)	0.095	2.21* (1.03-4.76)	0.042	1.62 (0.72-3.63)	0.239	1.61 (0.72-3.63)	0.248
Medicare, Medicaid & Private	23	0.92 (0.25-3.37)	0.894	0.72 (0.15-3.52)	0.681	0.84 (0.17-4.04)	0.824	0.86 (0.18-4.16)	0.853
Medicare, Medicaid & Military	5	-	-	-	-	-	-	-	-
Medicaid, Military & Private	0	-	-	-	-	-	-	-	-
Medicare, Medicaid, Military & Private	3	-	-	-	-	-	-	-	-
No Insurance	120	0.43* (0.20-0.92)	0.030	0.40 (0.15-1.06)	0.066	0.43 (0.15-1.21)	0.112	0.44 (0.16-1.25)	0.123

† Model 1: Adjusted for Age and Hearing Loss
‡ Model 2: Adjusted for Age, Hearing Loss, Gender, Race, Education, and Income
†† Model 3: Adjusted for Age, Hearing Loss, Gender, Race, Education, Income, Hypertension, Diabetes, Stroke & Smoking
* $p < 0.05$
