# Supplementary material for: Health insurance status and hearing aid utilization in U.S. older adults: A population-based cross-sectional study
Source: PLoS One. 2026 Jan 27;21(1):e0341570. doi: 10.1371/journal.pone.0341570 (PMC12843536; doi:10.1371/journal.pone.0341570)
Supplement: S4 Table — (PDF) [file pone.0341570.s004.pdf]

S4 Table. Logistic regression analysis examining the association between mutually exclusive insurance coverage and regular hearing aid use

Insurance Coverage	Cohort Size (n)	Unadjusted		Multivariable Models					
		OR (95% CI)	P-Value	Model 1† OR (95% CI)	P-Value	Model 2‡ OR (95% CI)	P-Value	Model 3†† OR (95% CI)	P-Value
Medicare only	954	1 (ref)	-	1 (ref)	-	1 (ref)	-	1 (ref)	-
Military only	24	3.01 (0.96-9.42)	0.058	3.52 (0.91-13.71)	0.069	3.59 (0.82-15.76)	0.090	3.64 (0.82-16.17)	0.0.90
Medicaid only	45	0.48 (0.11-2.10)	0.331	0.28 (0.05-1.64)	0.159	0.31 (0.04-2.29)	0.250	0.34 (0.05-2.35)	0.272
Private only	216	0.88 (0.47-1.62)	0.673	1.19 (0.56-2.51)	0.651	1.14 (0.53-2.46)	0.731	1.13 (0.53-2.42)	0.757
Military & Medicare	155	2.15* (1.27-3.63)	0.004	2.16* (1.12-4.14)	0.021	2.02* (1.03-3.94)	0.040	2.10* (1.07-4.11)	0.031
Medicaid & Medicare	205	0.31* (0.13-0.74)	0.008	0.21* (0.08-0.58)	0.002	0.40 (0.14-1.13)	0.083	0.41 (0.14-1.16)	0.092
Private & Medicare	1,355	1.54* (1.16-2.05)	0.003	1.80* (1.27-2.55)	0.001	1.44 (0.99-2.08)	0.050	1.43 (0.99-2.07)	0.056
Military & Medicaid	1	-	-	-	-	-	-	-	-
Military & Private	8	2.41 (0.43-13.36)	0.314	2.50 (0.23-26.84)	0.449	2.67 (0.23-30.60)	0.428	2.61 (0.24-28.47)	0.432
Medicaid & Private	10	1.61 (0.32-8.09)	0.566	1.67 (0.25-11.31)	0.600	2.04 (0.28-14.79)	0.482	2.08 (0.29-14.97)	0.466
Medicare, Private & Military	48	1.85 (0.86-3.98)	0.114	1.91 (0.78-4.67)	0.156	1.31 (0.51-3.37)	0.568	1.33 (0.51-3.43)	0.560
Medicare, Medicaid & Private	23	0.48 (0.06-3.81)	0.489	0.26 (0.02-2.77)	0.265	0.40 (0.04-3.94)	0.433	0.42 (0.04-4.11)	0.454
Medicare, Medicaid & Military	5	-	-	-	-	-	-	-	-
Medicaid, Military & Private	0	-	-	-	-	-	-	-	-
Medicare, Medicaid, Military & Private	3	-	-	-	-	-	-	-	-
No Insurance	120	0.42* (0.16-1.08)	0.073	0.40 (0.11-1.42)	0.159	0.50 (0.13-1.94)	0.320	0.51 (0.13-2.01)	0.337

† Model 1: Adjusted for Age and Hearing Loss
‡ Model 2: Adjusted for Age, Hearing Loss, Gender, Race, Education, and Income
†† Model 3: Adjusted for Age, Hearing Loss, Gender, Race, Education, Income, Hypertension, Diabetes, Stroke & Smoking
* $p < 0.05$
